# Supplementary material for: The ‘Candidatus Phytoplasma mali’ effector protein SAP11CaPm interacts with MdTCP16, a class II CYC/TB1 transcription factor that is highly expressed during phytoplasma infection
Source: PLoS One. 2022 Dec 15;17(12):e0272467. doi: 10.1371/journal.pone.0272467 (PMC9754288; doi:10.1371/journal.pone.0272467)

The ‘*Candidatus* Phytoplasma mali’ effector protein $SAP11_{CaPm}$ interacts with MdTCP16.

S1 Fig. Reference Sequence of XM_008376500.2. The sequence includes the CDS for XP_008374722.1 (*MdTCP16*) with TCP domain and the identified part in the Y2H screen. Graph was generated with Geneious R11.1.5.

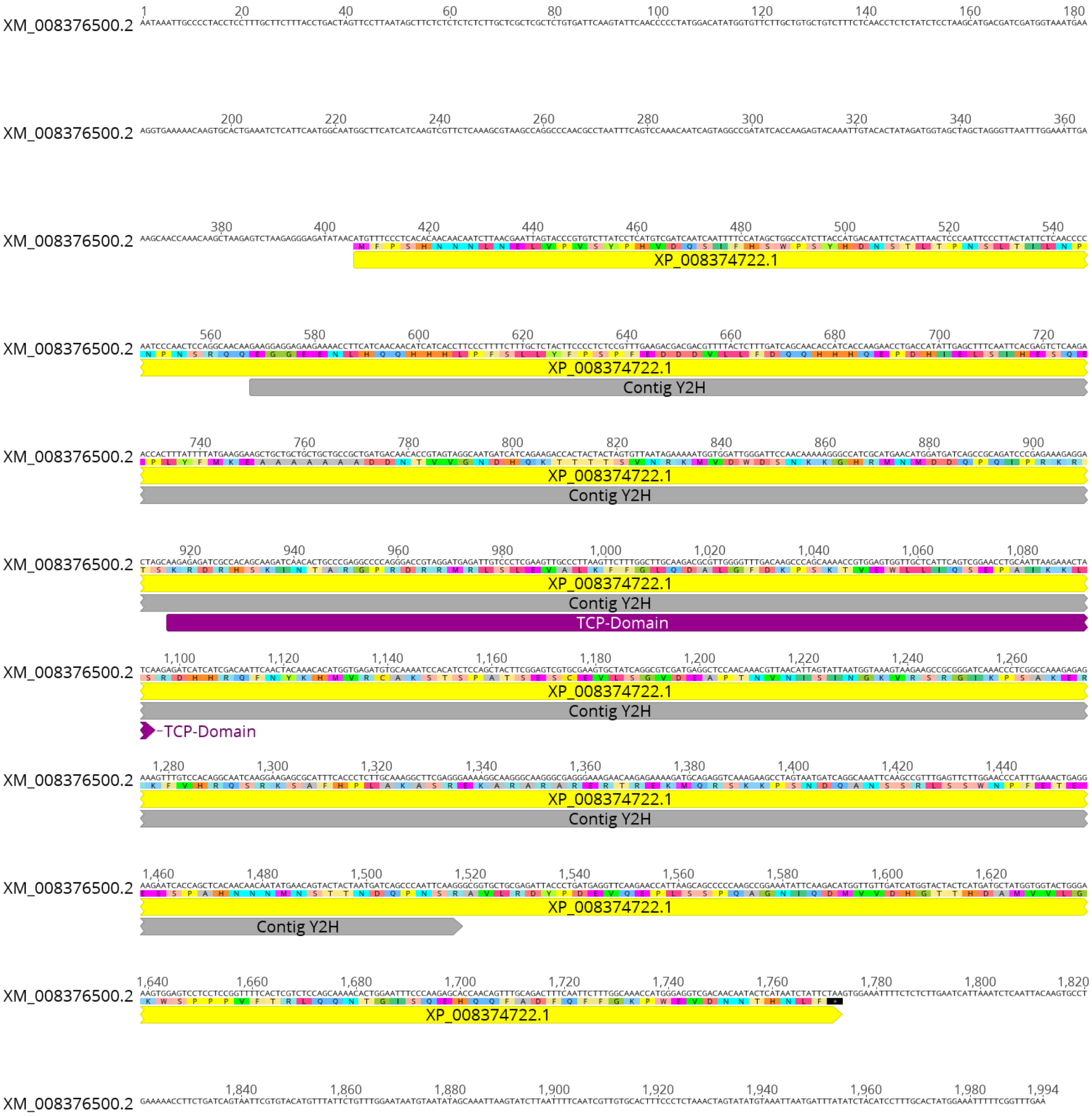

Supplement: S1 Fig — The sequence includes the CDS for XP_008374722.1 (MdTCP16) with TCP domain and the identified part in the Y2H screen. (PDF) [file pone.0272467.s001.pdf]
