# Supplementary material for: The ‘Candidatus Phytoplasma mali’ effector protein SAP11CaPm interacts with MdTCP16, a class II CYC/TB1 transcription factor that is highly expressed during phytoplasma infection
Source: PLoS One. 2022 Dec 15;17(12):e0272467. doi: 10.1371/journal.pone.0272467 (PMC9754288; doi:10.1371/journal.pone.0272467)

S2 Fig. Phytoplasma concentration of leaf and root samples. The normalized phytoplasma concentration is given as the ratio of the '*Ca*. P. mali' specific *16S* gene copies and the *Malus x domestica* single-copy gene *ACO.* Phytoplasma concentration was quantified in seven infected leaf samples from greenhouse plants (three from spring and four from autumn), in three naturally infected pooled leaf samples (one from spring, two from autumn) and in one naturally infected pooled root sample (autumn).

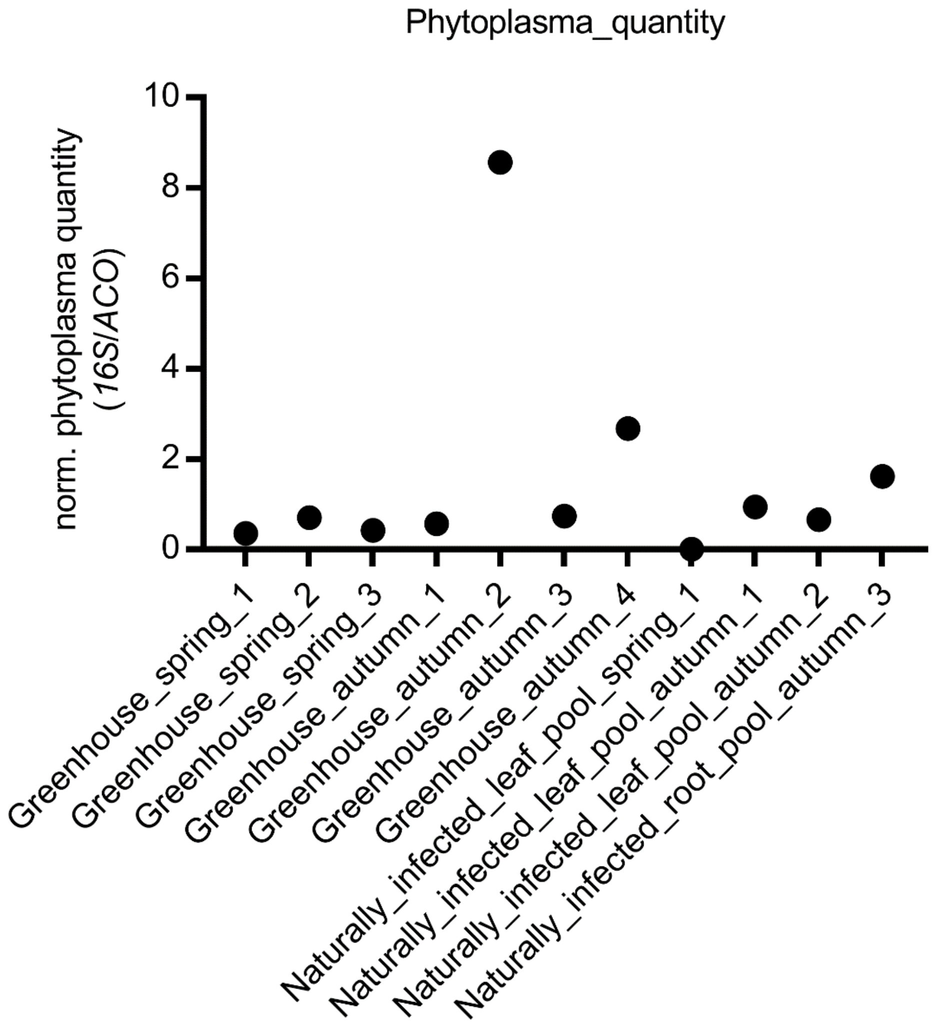

Supplement: S2 Fig — The normalized phytoplasma concentration is given as the ratio of the ’Ca. P. mali’ specific 16S gene copies and the Malus x domestica single-copy gene ACO. Phytoplasma concentration was quantified in seven infected leaf samples from greenhouse plants (three from spring and four from autumn), in three naturally infected pooled leaf samples (one from spring, two from autumn) and in one naturally infected pooled root sample (autumn). (PDF) [file pone.0272467.s002.pdf]
