# Supplementary material for: The ‘Candidatus Phytoplasma mali’ effector protein SAP11CaPm interacts with MdTCP16, a class II CYC/TB1 transcription factor that is highly expressed during phytoplasma infection
Source: PLoS One. 2022 Dec 15;17(12):e0272467. doi: 10.1371/journal.pone.0272467 (PMC9754288; doi:10.1371/journal.pone.0272467)
Supplement: S1 Table — Lowercase letters indicate bases for Gateway-attB site overhangs or SfiI restriction site overhangs. (PDF) [file pone.0272467.s003.pdf]

S1 Table. Primers used in this study.

**Name**	**Sequence**	**Reference**
ATP00189pP_B3	ggggacaactttgtataataaagttgctATGTCTCCTCCTAAAAAAGATT	This publication
ATP00189pP_B2	ggggaccactttgtacaagaaagctgggtaTTATTTTTTTCCTTTGTCTTTATTG	This publication
ATP00189pP_B2ns	ggggaccactttgtacaagaaagctgggtaTTTTTTTCCTTTGTCTTTATTGTT	This publication
MdTCP16_B1	ggggacaagtttgtacaaaaaagcaggcttaATGTTTCCCTCACACAACAAC	This publication
MdTCP16_B4	ggggacaactttgtatagaaaagttgggtgTTAGAATAGATTATGAGTATTGTT	This publication
MdTCP16_B4ns	ggggacaactttgtatagaaaagttgggtgGAATAGATTATGAGTATTGTTGT	This publication
MdTCP16_SfilA_fwd	ccccccggccattacggccATGTTTCCCTCACACAACAAC	This publication
MdTCP16_SFilB_rev	ccccccggccgaggcggccTTAGAATAGATTATGAGTATTGTT	This publication
qMdTCP16_fw	AAGATCAACACTGCCCGAGG	This publication
qMdTCP16_rv	GCGTCTTGCAAGCCAAAGAA	This publication
qMdTCP25_fw	ATTGTTCGATCCACCGGACG	This publication
qMdTCP25_rv	GAATTGGATGGCAGTGTGGG	This publication
qMdTCP24_fw	AGAAGGCCCTAGCAGTGGAA	This publication
qMdTCP24_rv	TCTTTCGACTTTGCTCGCCA	This publication
Md_GAPDH_fw	GTTCGTTGTTGGTGTGAACG	[1]
Md_GAPDH_rv	GTCTTCTGGGTGGCAGTGAT	[1]
Md_tip41_fw	ACATGCCGGAGATGGTGTTTGG	[2]
Md_tip41_rv	ACTTCCAGAGTACGGCGTTGTG	[2]
Md_EF1α_fw	ACTGTTCCTGTTGGACGTGTTG	[2]
Md_EF1α_rv	TGGAGTTGGAAGCAACGTACCC	[2]

Lowercase letters indicate bases for Gateway-*attB* site overhangs or *SfiI* restriction site overhangs.

[1] Guerriero G, Giorno F, Ciccotti AM, Schmidt S, Baric S. A gene expression analysis of cell wall biosynthetic genes in *Malus x domestica* infected by '*Candidatus* Phytoplasma mali'. Tree Physiol. 2012; 32:1365–77. doi: 10.1093/treephys/tps095 PMID: 23086810.

[2] Giorno F, Guerriero G, Biagetti M, Ciccotti AM, Baric S. Gene expression and biochemical changes of carbohydrate metabolism in in vitro micro-propagated apple plantlets infected by 'Candidatus Phytoplasma mali'. Plant Physiol Biochem. 2013; 70:311–7. doi: 10.1016/j.plaphy.2013.05.040 PMID: 23811119.
