# Supplementary material for: The ‘Candidatus Phytoplasma mali’ effector protein SAP11CaPm interacts with MdTCP16, a class II CYC/TB1 transcription factor that is highly expressed during phytoplasma infection
Source: PLoS One. 2022 Dec 15;17(12):e0272467. doi: 10.1371/journal.pone.0272467 (PMC9754288; doi:10.1371/journal.pone.0272467)
Supplement: Attachment — Submitted filename: Response to reviewers.pdf [file pone.0272467.s005.pdf]

Subject: Resubmission of PONE-D-22-20188R1

thank you very much for the valuable input and the comments regarding the previous version of our manuscript with the title "The '*Candidatus* Phytoplasma mali' effector protein SAP11CaPm interacts with MdTCP16, a class II CYC/TB1 transcription factor that is highly expressed during phytoplasma infection". Along the revised manuscript we provide point-by-point responses to every reviewer's concern. Every author's response to a reviewer's comment is indicated with arrows (>>>) and written in red. All changes that have been performed to the previous version of the manuscript are indicated by tracked changes. The manuscript text, figures, legends, or any other part of the previously submitted documents have not undergone any substantial changes if not requested by the reviewers.
We hope that our manuscript now fulfills all criteria for publication in PLOS ONE.

Reviewer #1: Dear authors,
Thank you very much for the revised version. All my raised comments were adequately addressed.
Good luck for your future work.

Reviewer #2: The authors have clarified most of the issues I raised during my last review. I still have a few points about Figure 3 that need to be addressed:

Figure 3C: Since the samples plotted here come from different growing conditions (naturally vs graft infected and greenhouse vs foil tunnel) and, as shown in panels A and B, have different expression profiles, they shouldn't be analyzed as a single set of samples. The expression level of MdTCP16 in grafted plants is more than ten times lower than the transcription level in naturally infected trees. Similarly, the correlation analysis should be performed separately.

>>> The correlation analyses shown in the former Fig 3C and Table 1 were changed according to the reviewer's suggestions. This improved the comprehensibility of the whole graph and thus the interpretation of the results. Expression levels were compared separately and regarding the growing conditions. The statistical correlation analysis was also performed on the separated sample sets as suggested. The adjusted analytical approach did not affect the overall results, i.e. the same results or trends were found in the Figures 3 C-D as in the former Fig 3C. The text in the manuscript was changed according to the changes in the analyses and the graph.

Figure 3D: Five grafted inoculated samples were plotted in figure 3B, but in figure 3D only three samples for spring and four for autumn were plotted. What is the rationale behind the exclusion of some of the samples?

>>> For the phytoplasma quantification in Fig 3D DNA was required whereas for the expression analyses RNA/cDNA was used. Since the phytoplasma concentration was determined in a second moment there was not enough material for a DNA extraction for every sample left. We tried a phytoplasma quantification based on cDNA but did not succeed. The comparison was performed as follows: In a pilot-study with a subset of samples we compared the phytoplasma quantification results using cDNA with those using genomic DNA from the same sample material. Summarizing the results that we got, we found that DNA-based quantification results did not correspond to those based on cDNA performed in parallel. We must admit that we did not further invest time in finding an appropriate phytoplasmal housekeeping gene that could have been used for accurate cDNA-based phytoplasma quantification. That's why we -unfortunately- cannot show results all ten samples in Fig 3D. To avoid appearing untransparent in this regard, we now mention the reason for the discrepancy in greenhouse sample numbers between 3B and 3F (before 3D) in the figure legend.
